# Coping with Everyday Stress, Assessment and Relationships, Psychological Adjustment, Well-Being and Socioemotional Adjustment

**DOI:** 10.3390/ejihpe12080068

**Published:** 2022-08-01

**Authors:** Francisco Manuel Morales Rodríguez

**Affiliations:** Department of Educational and Developmental Psychology, Faculty of Psychology, Campus Universitario de Cartuja, University of Granada, 18071 Granada, Spain; fmmorales@ugr.es

The use of effective or functional coping strategies guarantees quality of life and psychological well-being in different stages of development. Notably, the strategies for coping with the everyday stress that a specific problem or situation creates differ from those that need to be implemented in another problem or situation. With the current situation caused by the coronavirus disease 2019 pandemic, research related to the study of coping and its relationship with adjustment, maladjustment, and psychopathology is even more necessary.

Therefore, this Special Issue presents recent research that can be useful for the future design of psychoeducational actions and interventions to train effective strategies for coping with everyday stress in different contexts. For instance, the first article [1] expands on the changes in instruction and pedagogical practices due to the transformation of the academic system caused by coronavirus disease 2019. In particular, it focuses on how these changes contributed to increasing students’ anxiety levels and the need for interventions to support university students with safe learning environments and the promotion of strategies and counseling services for coping with the situation. Similarly, another study [2] focuses on evaluating the relevant construct of school anxiety in the adolescent population and indicators of psychological well-being, finding concerns regarding self-regulation of emotions in this population. Thus, it can be pointed out that more studies are needed to focus on variables such as school anxiety and daily stressors.

Another recent interesting study [3] compares differences in the relationship between coping strategies and psychological well-being in a sample of parents with sensory impairment and those of parents without these impairments. It is determined through the study that parents who are blind and deaf are more likely than parents without sensory loss to use the coping strategies of social support, avoidance, and turning to religion, which may affect their psychological well-being in one way or another. Similarly, another study [4] focuses on validating an instrument for a more accurate and reliable assessment of religious motivations, and future studies are expected to further investigate this construct.

Regarding the situation of the pandemic, Rodríguez et al. [5] carried out relevant research in Spain. The objective of their study was to analyze the stress experienced owing to the restrictions and adaptations resulting from the coronavirus disease 2019 pandemic. Notably, they considered the sociodemographic characteristics of a sample of 1269 individuals aged between 18 and 70 years, mostly women, to whom they administered an instrument to assess the daily stress perceived in that situation. Among other results, they found that individuals who perceived higher stress levels during confinement were those with low income, women, and those aged below 25 years. The results also showed that individuals who could combine face-to-face and remote work experienced less stress. The evidence obtained in this study, one of the first studies in the Spanish context, is very relevant for designing future actions for prevention, diagnosis, and treatment.

Another study [6] introduces innovative elements in the field of coping with the presentation of an educational intervention focused on positive massage for improving well-being and communication to help prevent stress. Similarly, another innovative subject, highly topical in the academic context and related to health, given its numerous applications for improving well-being and quality of life, is that of coping and mindfulness. Marais et al. [7] designed a successful intervention program based on mindfulness in which members of a French research department participated. They found that after the program, participants in the intervention group experienced an increase in their psychological flexibility, effective time management, well-being, and mental health compared to participants in the control group.

A psychological construct that can be key when talking about stress prevention in the school context, which is one of the most stressful contexts impacting daily life, is resilience, which is addressed in the study by Romano et al. [8]. They analyze the relationships between school engagement, teacher emotional support, and academic resilience in a sample of 205 Italian secondary school students (aged between 14 and 19 years, with a nearly gender-balanced sample). The results show a relationship between teachers’ perceived emotional support and academic resilience. They also found statistically significant associations between academic resilience and emotional support perceived by teachers and school engagement. This work shows that resilience is vital in promoting the well-being and adaptation of students in the school context.

In recent years, there has been growing interest in studying the coping process and its evaluation. Currently, many works related to the topic can be found. The increasing attention being given to the theme of coping is possibly caused by its potential practical applications. This is because coping has to be present in the face of the continuous challenges that human beings experience. Daily concerns in childhood and adolescence have consequences beyond the academic life of students, having direct repercussions on family-, school-, social-, and health-related life. Moreover, based on psychology, the study of coping is applied to diagnosing and treating diseases, such as depression, anxiety, chronic illness, and cancer. Training productive or functional coping strategies has become especially noticeable in the pandemic. The studies presented in this Special Issue provide relevant information for continued advancement in this field and contribute to carrying out psychoeducational intervention programs with efficacy and efficiency aimed at stress-coping training. Notably, more studies are needed to establish robust theoretical models in which the role of certain variables and their explanatory capacity in the differential use of different coping strategies in varied social, educational, and health-related contexts can be analyzed.
